# Pregnancy in Patients with Type One Diabetes Mellitus Treated with Continuous Subcutaneous Insulin Infusion—Preconception Basal Insulin Dose as a Potential Risk Factor for Fetal Overgrowth?

**DOI:** 10.3390/ijerph17186566

**Published:** 2020-09-09

**Authors:** Gloria Lekšić, Maja Baretić, Marina Ivanišević, Dubravka Jurišić-Eržen

**Affiliations:** 1 Department of Internal Medicine, University Hospital Centre Zagreb, 10000 Zagreb, Croatia; gleksic@gmail.com; 2 Division of Endocrinology and Diabetes, Department of Internal Medicine, University Hospital Centre Zagreb, 10000 Zagreb, Croatia; 3 School of Medicine, University of Zagreb, 10000 Zagreb, Croatia; marina.ivanisevic@pronatal.hr; 4 Department of Gynaecology and Obstetrics, University Hospital Centre Zagreb, 10000 Zagreb, Croatia; 5 Division of Endocrinology, Diabetes and Metabolism, Department of Internal Medicine, Clinical Hospital Centre Rijeka, 51000 Rijeka, Croatia; dubravka.jurisic.erzen@gmail.com; 6 School of Medicine, University of Rijeka, 51000 Rijeka, Croatia

**Keywords:** type one diabetes mellitus, pregnancy, preconception, insulin pump, large-for-gestational-age neonates

## Abstract

Despite widespread use of technology, type one diabetes mellitus (T1DM) is still a great clinical challenge during pregnancy. This study aims to assess how prenatal variables of T1DM patients using continuous subcutaneous insulin infusion (CSII) influence pregnancy outcomes. We performed a retrospective study of 35 patients with T1DM treated with CSII during pregnancy. Alterable preconception variables (A1C, body mass index, basal and bolus insulin dose) were analysed as possible contributors to birth weight and large-for-gestational-age (LGA) prevalence. Inclusion criteria were presence of T1DM for more than two years, A1C < 7.4% and treatment with CSII for at least three months prior to conception. The preconception basal insulin dose and A1C had a significant correlation to the neonatal birth weight (*p* = 0.01, r = 0.4 and *p* = 0.04, r = 0.3, respectively) and were significant in regression analysis together contributing 22% of the variance in birth weight percentiles (sig = 0.17, R square = 0.22). Prevalence of LGA was 46%. Women who had LGA neonates also had a higher preconception basal insulin dose compared to women with non-LGA neonates (26 ± 9 vs. 18 ± 7 IU (international units), *p* = 0.01). The LGA group had a higher preconception A1C, but it did not reach statistical significance (6.5 ± 0.5% vs. 6.2 ± 0.9%, respectively, *p* = 0.2). Women with T1DM treated with CSII who had unregulated glycaemia and more basal insulin were at greater risk for development of LGA neonates.

## 1. Introduction

Type I diabetes mellitus (T1DM) affects about 0.1–0.2% of all pregnancies [1]. It increases the risk of maternal and neonatal complications; miscarriage, preeclampsia, macrosomia, large-for-gestational age neonates (LGA), neonatal hypoglycaemia, etc. Hyperglycaemia in pregnancy correlates with foetal blood glucose level; it is recognised as a major teratogen [2].

Treatment of T1DM is currently experiencing significant improvements due to technological advancement; there are plenty of innovative methods developed for blood glucose measurements as well as for insulin delivery [3,4]. Continuous glucose monitoring systems (CGM) use an electrochemical enzymatic sensor that measures glucose in interstitial fluid at regular intervals. They provide a continual display of measured glucose on the monitor of a device or mobile phone and are equipped with alarms signalling hypo- or hyperglycaemia. Insulin pump, i.e., continuous subcutaneous insulin infusion (CSII) is a device that delivers insulin at a constant rate, therefore imitating physiological insulin secretion that is particularly helpful in demanding cases such as pregnancy. Artificial pancreas integrates CGM and insulin pump with a closed loop system. The data from CGM provide blood glucose readings and the insulin pump automatically adjusts the basal insulin rate using a special algorithm. An artificial pancreas is able to autonomously stop insulin when hypoglycaemia is detected and to restart insulin delivery when glucose levels recover [3,4].

Despite widespread use of technology, T1DM is still a great clinical challenge during pregnancy, from conception to the breastfeeding, where insulin requirements are constantly changing [5]. Rapid, flexible and precise dosing of CSII seems to be the optimal solution since it can release small doses of insulin continuously (basal insulin) and a user-initiated bolus dose at mealtime [1,3,4]. In T1DM patients, CSII is usually started before conception [2,3].

Some outcomes from T1DM pregnancies have significantly improved over the last decade. However, LGA prevalence is increasing and it is still an unresolved issue [6,7,8]. LGA neonates are associated with complications during labour and with neonatal morbidity [8,9,10]. It is also a risk factor for future obesity and type 2 diabetes [11].

So far, there are no sufficient data on preconception markers and/or treatment patterns associated with pregnancy outcomes in women with T1DM on CSII therapy [10].

This study aimed to assess how some alterable prenatal variables may influence pregnancy outcomes in women with T1DM using CSII.

## 2. Patients and Methods

A total of 297 pregnant patients with T1DM were followed at the University Hospital Centre in Zagreb (State Referral Centre for treatment of diabetes in pregnancy). Only 42 of them (14%) were treated with CSII from conception to delivery. Data of 35 patients who met the inclusion criteria were retrospectively analysed. Inclusion criteria were diagnosis of T1DM for at least 2 years before pregnancy, A1C < 7.4% and treatment with CSII (Medtronic MiniMed™ Paradigm™ Veo™) for at least 3 months prior to conception.

Analysed records included anamnestic data (age at T1DM diagnosis, age at CSII setup, age at the time of conception), anthropometric measurements (body mass index—BMI) and variables associated with glucose regulation (A1C, total daily insulin dose, daily basal and bolus insulin dose, basal/bolus insulin ratio). Pregnancy outcomes were gestational weight gain, neonatal birth weight and length, week of delivery, APGAR (10—completely healthy newborn; below 5—concerning health of a newborn) score, presence of neonatal hypoglycaemia, prevalence of LGA and macrosomia. LGA neonates were defined as birth weight >90th percentile for gestational age and sex. Macrosomia was defined as birth weight > 4000 g.

Pregnancies were divided into two groups: planned and unplanned, where all the parameters (preconception variables and pregnancy outcomes) were analysed in both groups.

Preconception period was defined as a timeframe of three months prior to conception. Since all the patients had frequent ambulant follow-ups, preconception data were available even for unplanned pregnancies.

Data presented in this manuscript are preliminary results of the greater study which is still ongoing, and it follows pregnant women with T1DM at the University Hospital Centre in Zagreb from 2015.

The study was conducted in accordance with the Declaration of Helsinki, and the protocol was approved by the Ethics Committee of University Hospital Centre in Zagreb.

## 3. Statistical Analysis

Using the Shapiro–Wilk test, data distribution was defined as normal. The data were given as mean and standard deviation and categorical data as percentage (%). Alterable preconception variables that were analysed as possible contributors to the neonatal birth weight and LGA prevalence were A1C, BMI, basal and bolus insulin dose. Bivariate correlations with the Pearson coefficient and multivariate linear regression analysis were used to assess possible correlations and associations between selected preconception variables and neonatal birth weight.

An independent samples t-test was used to compare two groups of data.

Statistical analysis was performed using IBM SPSS Statistics 21, with the level of statistical significance set at *p* < 0.05.

## 4. Results

At the time of conception, mean age was 28 ± 2 years, age at T1DM diagnosis 14 ± 5 years and age at CSII setup 24 ± 4 years. The mean BMI was 24 ± 3 kg/m^2^ and 26% (9/35) of patients were overweight. Patients who were overweight before pregnancy had higher preconception A1C than patients with normal preconception BMI (6.6 ± 0.9% vs. 6.2 ± 0.6%, respectively). That difference did not reach statistical significance (*p* = 0.3). Other preconception characteristics are presented in Table 1.

Pregnancy was planned in 74% (26/35) of the cases. When comparing planned to unplanned pregnancies, there was a statistically significant difference in gestational weight gain (11.3 vs. 14.1 kg, respectively, *p* = 0.008). Mean preconception A1C in the group of planned pregnancies was 6.2 ± 0.8% and in the group of unplanned pregnancies, it was 6.5 ± 0.4% There was no statistically significant difference in other preconception parameters and pregnancy outcomes between two groups (BMI, basal and bolus inulin dose, total daily insulin dose, birth weight, length, LGA prevalence, macrosomia prevalence).

Pregnancy outcomes are shown in Table 2.

Preconception A1C, BMI, basal and bolus insulin dose were correlated to the neonatal birth weight (*p* = 0.04, r = 0.3; *p* = 0.6, r = 0.1; *p* = 0.01, r = 0.4, *p* = 0.08, r = 0.3, respectively) (Table 3). A1C and basal insulin dose had the strongest correlation to birth weight and were furthermore significant in multivariate linear regression analysis together contributing 22% of the variance in birth weight percentiles (sig = 0.17, R square = 0.223). There was no statistically significant difference in birth weight according to sex variable (*p* = 0.82).

**Figure 1:**
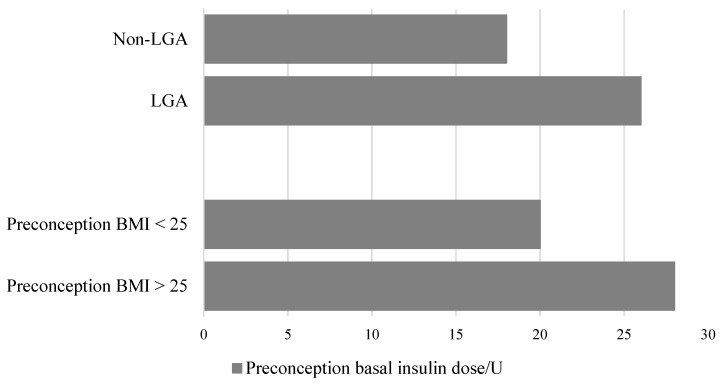
Preconception basal insulin dose in the group of non-LGA and LGA neonates. Preconception basal insulin dose in the group of normal-weight (BMI < 25 kg/m^2^) and overweight (BMI > 25 kg/m^2^) women. BMI = body mass index, LGA = large-for-gestational-age neonates, Non-LGA = non-large-for-gestational-age neonates, including appropriate-for-gestational-age and small-for-gestational-age neonates.

Prevalence of LGA neonates was 46% (16/35). The independent samples t-test was used to compare preconception basal insulin dose and preconception A1C in LGA and non-LGA group of pregnancies. The LGA group had a higher preconception basal insulin dose (26 ± 9 vs. 18 ± 7 IU, *p* = 0.01) (Figure 1).

Although the LGA group had a higher preconception A1C when compared to the non-LGA group, it did not reach statistical significance (6.5 ± 0.5% vs. 6.2 ± 0.9%, respectively, *p* = 0.2).

Preconception basal insulin dose and A1C were also compared in different preconception BMI groups. Overweight patients (BMI > 25 kg/m^2^) had higher preconception basal insulin dose than the group with normal BMI (<25 kg/m^2^); 28 ± 10 vs. 20 ± 8 IU, *p* = 0.02 (Figure 1). Preconception A1C was higher in the overweight group when compared to the group of normal weight patients, but it did not reach statistical significance (6.6 ± 0.9 vs. 6.2 ± 0.6%, respectively; *p* = 0.3).

## 5. Discussion

Our results yielded two preconception variables associated with foetal overgrowth in T1DM pregnancies treated with CSII. The first one is suboptimal preconception glucoregulation, an observation already proven in other studies [8,9,10]. The second one, a novelty, is a higher preconception basal insulin dose.

Regarding measures of glycaemia that predispose to the development of LGA neonates in T1DM pregnancies, there is some conflicting literature. Some research associated LGA neonates with poor glycaemic control in the third trimester of pregnancy [10,11,12] and the others with unregulated glycaemia at preconception/periconception period [8,9,10]. The preconception period is still a matter of controversy [9,10]. The critical time of metabolic shifts from mother to the foetus is unknown. Some studies reported that foetal hyperinsulinemia, which is the main promoter of “foetal adiposity”, starts very early in pregnancy. There is a possibility that it happens even before pregnancy occurs [13,14]. The effect of pre-pregnancy metabolic changes on the foetal development may be mediated through the modification of oocyte metabolism when an early setup of foetal hyperinsulinemia leads to the inability to reverse foetal growth in late pregnancy [13,14]. This could explain high LGA rates despite optimal glucoregulation in the third trimester. In our study, almost half of the pregnancies ended with LGA neonates, (46%), which is similar to the other larger studies where LGA prevalence ranged from 47 to 63% [1,6,9].

Treatment of T1DM during pregnancy on CSII requires specific modifications of basal/bolus insulin ratio because of fluctuating hormonal changes throughout gestation. In the first trimester (and preconception) the recommended basal/bolus ratio is 50/50 and in the last trimester it is 30−40/60−70 [15]. Basal insulin dose is usually calculated by the formula “basal insulin = 0.5 × total daily insulin dose”, yet some studies on patients using CSII, proposed that this formula overestimates basal insulin dose. They suggested that only 30% of basal insulin in T1DM patients is sufficient for achieving normoglycemia [16,17]. Our study associated higher preconception basal insulin dose with higher prevalence of LGA neonates.

Moreover, we reported on higher doses of basal insulin among overweight mothers when compared to the group of women with normal preconception BMI. Only few other studies reported on this topic; they associated higher dose of basal insulin with elements of metabolic syndrome (higher BMI and higher proportion of fat) in T1DM patients [18,19].

Furthermore, preconception BMI did not have a significant correlation on the neonatal birth weight which opposes some other studies where higher preconception BMI contributed to foetal overgrowth [20]. Still, the effect of maternal body weight might be stronger among obese patients. In our study, there were no obese pregnant women, 26% (9/35) were overweight.

When compared to the unplanned pregnancies, planned pregnancies had less gestational weight gain. A possible explanation is that planned pregnancies had a better adherence to therapy, suggesting that structured preconception counselling in such high-risk populations should not be neglected as a step toward positive pregnancy outcomes.

## 6. Conclusions

Suboptimal glycaemia and higher doses of basal insulin in preconception period are possible risk factors for development of LGA neonates in T1DM pregnancies treated with continuous subcutaneous insulin infusion.

Limitations of the study are the small sample size and the observational and retrospective design of this research. This could significantly influence conducted statistical tests.

Strengths of the study are strict inclusion criteria and standardised ambulatory follow-ups; therefore insulin changes were not confounded by maternal poor glycaemic control or variable insulin delivery (often seen at initiation of continuous subcutaneous insulin infusion).

## 7. Ethics Statements

The study was conducted in accordance with the Declaration of Helsinki, and the protocol was approved by the Ethics Committee of University Hospital Centre in Zagreb in July 2020.

Ethics approval number is 02/21 AG.

Participants were introduced with a Study Information Summary and signed consent for the study. The manuscript does not contain any individual person’s data in any form.

## Figures and Tables

**Table 1:** Preconception characteristics of patients with type one diabetes mellitus treated with continuous subcutaneous insulin Infusion.

Preconception Characteristics	
A1C/%	6.3 ± 0.8
A1C/mmol/mol	45 ± 0.8
TDD/units	40.3 ± 16.4
Basal insulin/IU	21.9 ± 9.4
Bolus insulin/IU	18.3 ± 8.6
Basal insulin/percentage	55 ± 9
Bolus insulin/percentage	45 ± 9
Body weight/kg	65.4 ± 8.1
BMI/kg/m^2^	24 ± 3

All values are expressed as mean and standard deviation. A1C = glycated haemoglobin, TDD = total daily insulin dose, BMI = body mass index, kg = kilograms, cm =centimetres, IU = international units.

**Table 2:** Pregnancy outcomes in patients with type one diabetes mellitus treated with continuous subcutaneous insulin infusion.

Pregnancy Outcomes	
Gestational weight gain/kg	12 ± 3
Birth weight/g, birth length/cm	3571 ± 695, 50 ± 2
Week of delivery and Apgar score	38 ± 1, 10 ± 1
LGA prevalence/%	46 (16/35)
Macrosomia prevalence/%	28 (10/35)
Neonatal hypoglycaemia/%	6 (2/35)

All values are expressed as mean and standard deviation. LGA = large-for-gestational-age neonates.

**Table 3:** Alterable preconception variables in correlation to the neonatal birth weight (bivariate correlations).

Preconception	*p*	r
A1C	0.04	0.3
Basal insulin dose/IU	0.01	0.4
Bolus insulin dose/IU	0.08	0.3
BMI/kg/m^2^	0.60	0.1

r = Pearson coefficient (measure of the strength of the association), *p* = significance of correlation (*p* < 0.05 is considered to be statistically significant).
